# Drooling: analysis and evaluation of 31 children who underwent bilateral submandibular gland excision and parotid duct ligation

**DOI:** 10.1016/S1808-8694(15)31120-4

**Published:** 2015-10-20

**Authors:** Dayse Manrique, Osiris de Oliveira Camponês do Brasil, Hugo Ramos

**Affiliations:** 1 PhD in Medicine - UNIFESP-EPM, Professor at the ENT Department - UNIFESP; 2 PhD. Postgraduate course advisor - UNIFESP-EPM; 3 PhD student - UNIFESP-EPM, AACD (Association to Care for Children with Disabilities) Otorhinolaryngologist

**Keywords:** salivary glands, neuropathy, cerebral palsy, drooling, surgical procedure

## Abstract

**Summary:**

Aim: To evaluate the safety of bilateral submandibular gland excision and parotid duct ligation in order to control drooling in children; to assess its long-term efficacy and complications.

**Study design:**

longitudinal cohort.

**Materials and Methods:**

Thirty-one children aged 6 to 13 years (7.6 years old in average), with multiple neurological disabilities were submitted to a bilateral submandibular gland excision with parotid duct ligation in order to control ptyalism between December 1999 and December 2005, mean follow up of 36 months.

**Results:**

According to Wilkie's success criteria, 87% of children had excellent or good results and insignificant morbidity was insignificant; with temporary parotid edema as the major complication.

**Conclusion:**

Bilateral submandibular gland excision with parotid duct ligation were safe to be performed in children, with 87% of success in drooling control.

## INTRODUCTION

Until very recently, recommended treatment for sialorrhea associated to cerebral palsy or mental retardation included speech therapy1, anti-cholinergic drugs^2^, tympanic neurectomy^3^,^4^, radiotherapy on salivary glands5 and parotid ducts repositioning towards tonsil fossae together with submandibular gland resection (Wilkie's procedure)^6^, ^7^, ^8^; and the Wilkie's procedure modification, which consists in the ligature of parotid ducts (PT) together with submandibular gland resection (SM)^9^, ^10^, ^11^. More recently, type A Botulin toxin (BTA) has been used in order to reduce saliva production, with temporary results.^12^,^13^

In order to reduce saliva production in patients with salivary fistulas and recurrent parotiditis, successful PT duct ligation has been reported^14^, ^15^. In 1978, some authors^9^ reported success in the ligature of PT ducts together with SM gland resection, describing an easier technique and less failure, in agreement with the authors of a study involving 79 children, about sialorrhea control.^10^

Patients with sialorrhea do not complain of excessive saliva production, but rather the incapacity to swallow it properly. Cineradiographic studies showed that while pharyngeal and esophageal swallowing phases have a normal pattern, voluntary muscle movements in the anterior two thirds of the tongue are disorganized^6^.

## OBJECTIVE

To study the success of bilaterally resecting the submandibular glands and ligating the parotid ducts in order to control sialorrhea, and the middle and long run consequences of saliva reduction.

## METHOD

Thirty one children followed at the Clínica de Otorrinolaringologia da AACD - São Paulo (Associação de Assistência à Criança Deficiente-AACD) underwent surgical treatment to control sialorrhea, from December of 1999 to December of 2005. This study was approved by the Research Ethics Committee of the AACD, under protocol # 06-2006. Age varied between 6 and 13 years, with average of 7.6 years. Etiologic diagnoses of the neurologic alteration were: cerebral palsy (15 patients), progressive chronic encephalopathy (2 patients), congenital myopathy (1 patient), acquired ischemic-anoxic encephalopathy (1 patient), Klinefelter's syndrome (1 patient), CNS malformation (10 patients), and Moebius sequence (1 patient).

**Figure 1:**
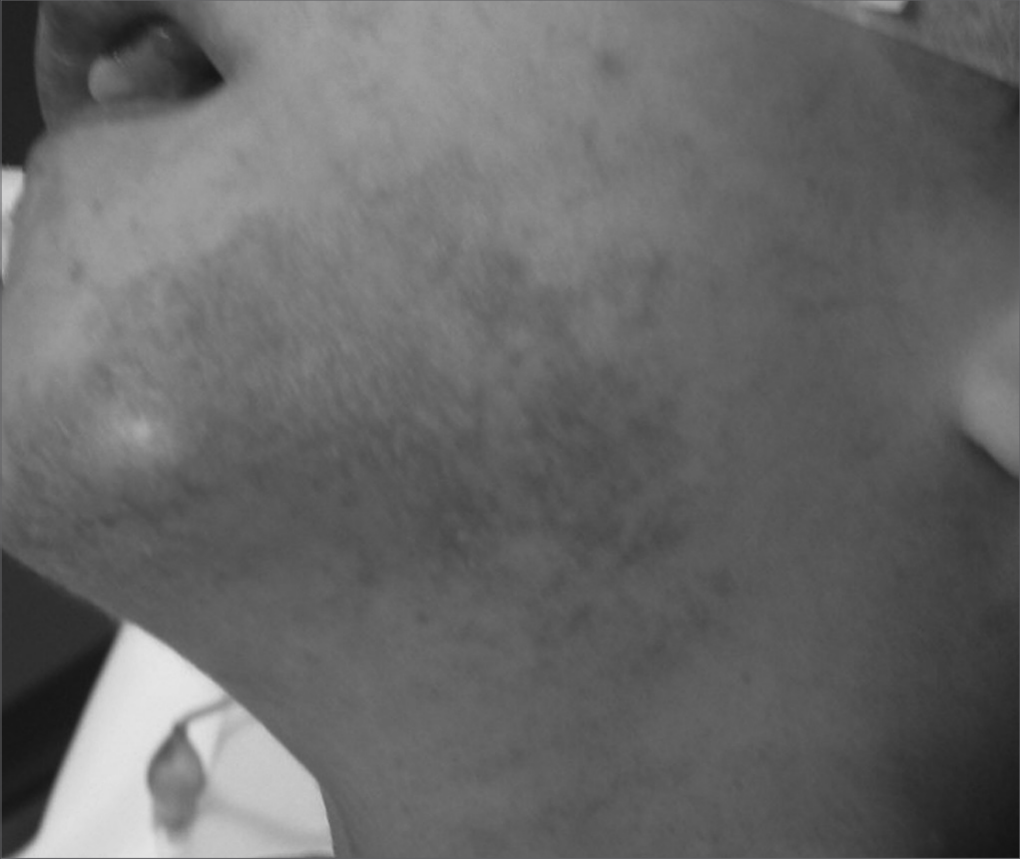
Chin dermatitis secondary to sialorrhea.

Study inclusion criteria were:

1. Minimum age: 5 years;
2. Prior treatment of upper air-digestive tract problems (mechanical obstruction, allergic causes, dental occlusion alterations);
3. Prior dental treatment;
4. Parents and family members' expectations and motivations concerning the surgical treatment;
5. Daily saliva volume requiring cups, napkins, towels, frequent clothes exchanges (more than four shirts per day), chin skin lesion secondary to saliva exposure (Figure 1); difficulty in performing hand tasks such as handling paper, canvas, toys, computers.

### Success criteria:

1. Eliminate the gauze, cup or towel that these patients carry with them, without adding other symptoms3.
2. The number of towels or napkins used in the postoperative, when compared to those used in the preoperative should be seen as a success indicator^8^.

Success criteria were subjectively assessed based on information provided by family members and care givers, according to Wilkie^6^.

Excellent: apparent normal saliva control.

Good: mild saliva oral escape, with or without saliva build up on the lips.

Reasonable: residual or thick saliva that bothers the patient.

Bad: control failure or feeling of dryness.

### Surgical technique

**Figure 2:**
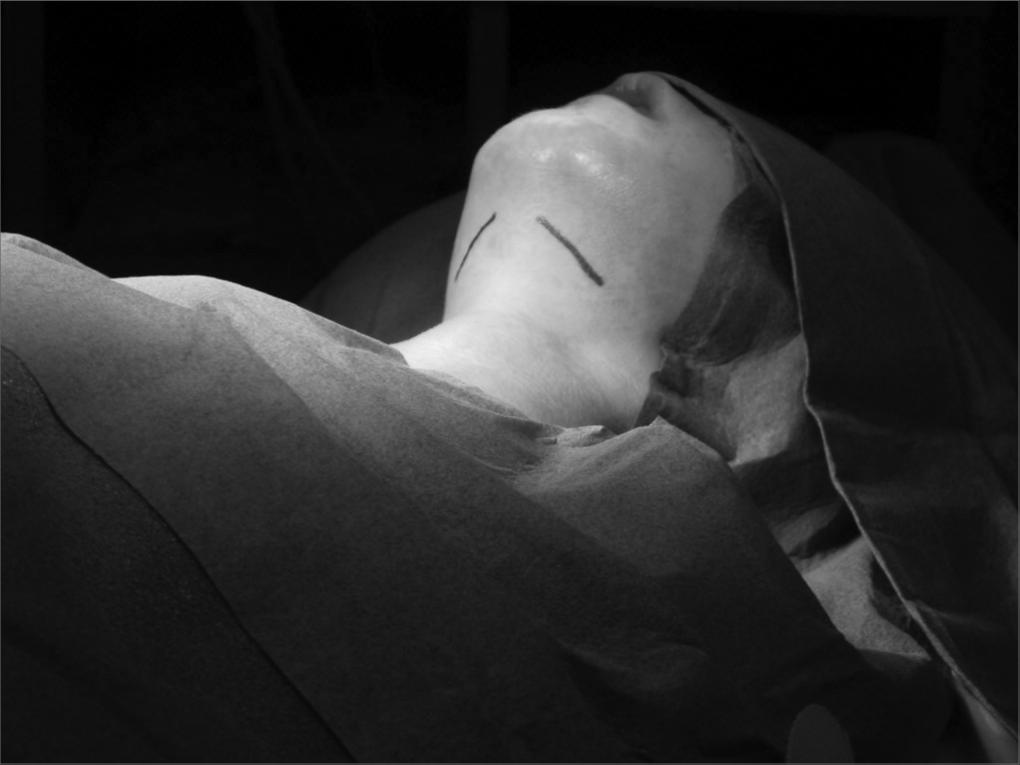
Incision topography for submandibular gland exeresis (submandibular region).

**Figure 3:**
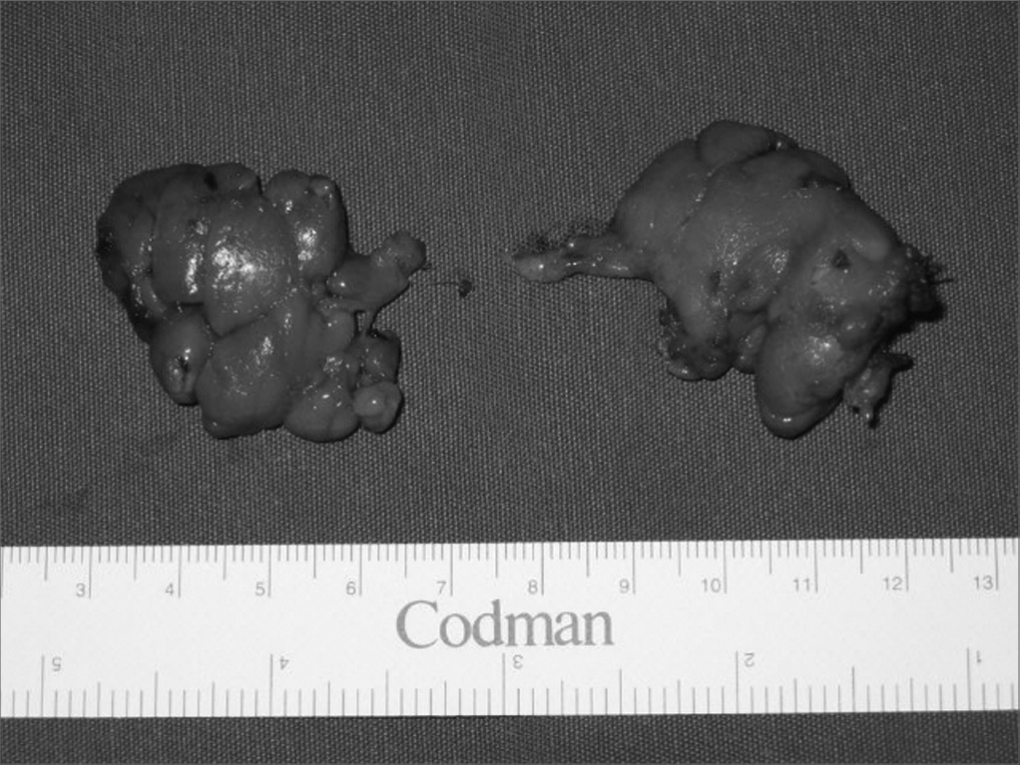
Excised right and left submandibular glands.

The operation is carried out in two phases, starting by the bilateral resection of the submandibular glands, with two independent incisions. One should proceed with a subcapsular dissection in order to avoid injuring the lingual and hypoglossal nerves. The mandibular branch of the facial nerve must be identified and spared. The SM gland duct and the secretory branch of the lingual nerve for the gland must be ligated; and the SM gland is removed. (Figures 2 and 3)

**Figure 4:**
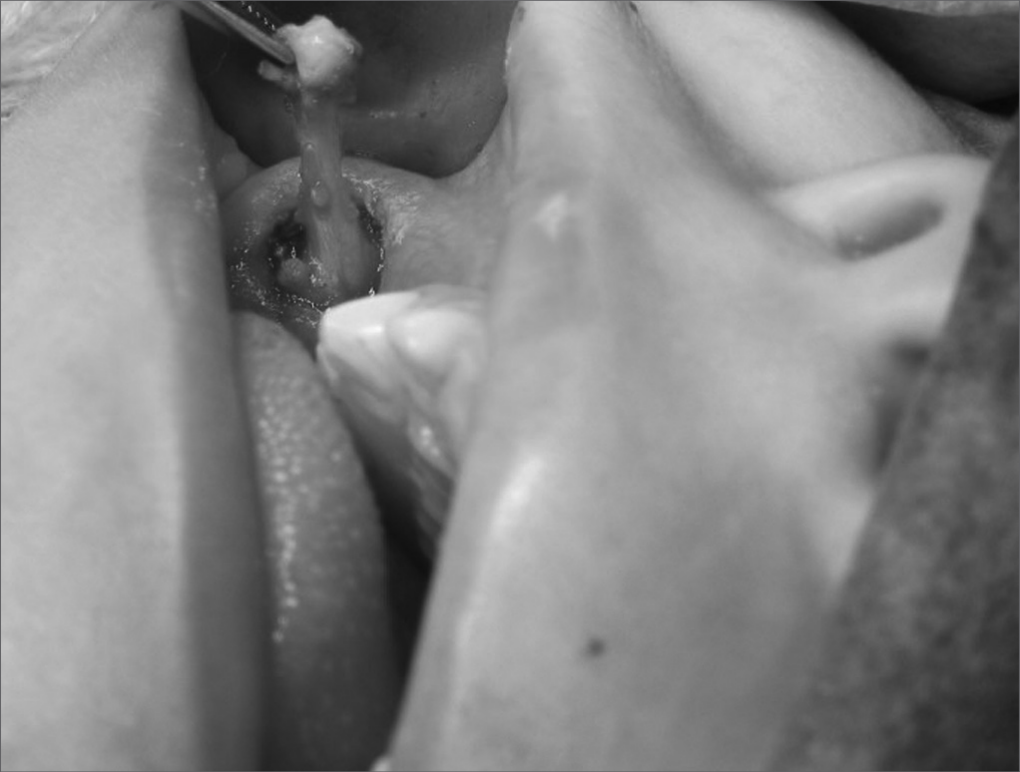
Right side parotid duct isolated after elliptical incision in the jugal mucosa.

The second phase is intraoral. One must identify the parotid duct hole (Stensen or Stenon duct), an elliptical incision is then made around the duct for 3mm of the mucosa that surrounds it, the PT duct is isolated in around 1cm, ligated in the proximal end, and the distal portion is resected. The jugal mucosa is brought together by means of suturing. (Figure 4)

## RESULTS

Thirty-one children underwent the aforementioned surgical treatment, 23 boys and 8 girls, with minimum age of 6 years and maximum age of 13 years, with average of 7.8 years.

Follow up time was of one year and six months, with average follow up time of 36 months. Follow up was bimonthly in the first year, every six months in the second year; and annually from then on.

Average hospital stay time was of 2 days, and the maximum hospital stay time was of 6 days (for one patient only, because of edema and mouth opening limitation).

The most frequent complication was an increase in parotid gland volume in the immediate postoperative time, which occurred in 25.8% of the patients. Such alteration did not have inflammatory characteristics, and in 48 hours it receded in all patients with the use of hormonal anti-inflammatory drugs.

There was unilateral parotid duct fistulization in 3 children (9.6%) observed in the jugal region, near the initial drainage site, which occurred in the first postoperative month and did not compromise procedure success, not requiring further interventions.

In two patients we observed a retention cyst formation in the late postoperative (two and three months after surgery) in the region neighboring the duct ligature. Punction was carried out then and a serous material oozed out, there was no recurrence.

In one patient, it was hard to chew solid dry food, and his meal was associated to fluid ingestion. Although we specifically surveyed and questioned about dental or periodontal problems there was no increase in the incidence of complaints or alterations in the average follow up period of 36 months.

According to Wilkie's criteria^6^ regarding procedure success, we found 27 children with either excellent or good postoperative results, making up 87% of the children; and no bad results.

## DISCUSSION

About 10% of the children with CP (cerebral palsy) present some important swallowing disorder^5^. Moreover, hundreds of patients with neurologic diseases have dysphagia and motor oral dysfunction in their ability to swallow saliva. There is considerable mismatch between problem magnitude and the amount of research carried out in an attempt to help these individuals. It is an alteration that involves social interaction and quality of life; and therapeutic options are very well accepted by this population, even those that offer temporary results. Saliva oral escape towards the chin or chest is stigmatizing, inadequately associated to cognitive alteration, being in fact oral motor control ability.

Objective methods to quantify saliva production require scintigraphy, which is the most specific and less invasive test to assess salivary gland function; nonetheless, we agree with other authors who state that it is not an essential exam and one that should be ordered routinely in order to indicate surgery^5^. Sialography is another method used to assess salivary gland function, but it requires gland catheterization, contrast injection and later radiological assessment. It is more invasive and a harder technique to be performed in children, and besides, it does not offer functional results.

Many options of clinical/drug treatments, type A Botulin toxin in the salivary glands and different surgical techniques have been reported for the treatment of sialorrhea in patients with swallowing disorders. Medication is based on the use of anticholinergic or anti-Parkinson drugs; however, these are not safe to be used in children, since they have side effects that may worsen their neurologic condition and easily cause tolerance. Botulin toxin in the salivary glands is able to block neuroglandular transmission, it is effective, though temporary, and bears high cost, but it may be an option in the elderly with advanced disease that bear high surgical risk. The choice of technique was based on the best results attained with PT duct ligation, previously reported by other authors, regardless of having to retroposition the parotid duct^9^,^14^. Many surgical techniques have explored the possibility of SM gland maintenance, sectioning gland innervation by the chorda tympani nerve and tympanic neurectomy, or resection of the submandibular ganglion; however, long term results are disappointing because reinnervation always happens. SM gland duct ligation may increase the incidence of salivary lithiasis due to salivary characteristics in these glands and because of the ascending positioning of the duct in relation to the oral cavity; and also the technical difficulty of isolating the submandibular ducts from the sublingual salivary glands ducts in children. There is no surgical technique better than the modified Wilkie procedure which was the one employed in this study; it is considered Gold Standard in sialorrhea control, although it is not very much discussed or used by otorhinolaryngologists in the rehabilitation of chronic patients. The increase in the prevalence of neurologic diseases, survival improvement in patients with the use of more recent drug developments and non-invasive mechanical ventilation, portable mechanical ventilation devices, have made the life quality complaint of these patients more promptly valued and treated.

We consider the results obtained in the middle and long run follow up as excellent or good in 87% of the children, making sialorrhea control satisfactory in these patients. Such result is compatible with literature reports.^10^ Favorable results were kept from the first to the last follow up, except in two children in whom there was a worsening in their neurologic condition and the results went from good to reasonable. In children with congenital or acquired oral function incapacity, surgical correction should be stimulated, especially in those above five years of age, in whom neurologic development is more stabilized and much gain from speech therapy is not expected.^1^,^7^

Surgical morbidity is low and average hospital stay time was of two days, in agreement with reports in the literature.10 The level of motor compromise should not influence surgical decision, having in mind that even totally dependent patients may benefit from the procedure. The complications found are treated clinically, and the most frequent one was the painful volume increase in the parotid region during immediate postop, which occurred in 8 children (25.8%). Other authors also observed this alteration; and also serum amylase increase for up to two weeks of postop, without inflammatory characteristics.^10^

The possible dental or periodontal alterations brought about by saliva reduction can not be isolated from the consequent repercussions of anticonvulsive drug actions, dental occlusion alterations, oral breathing, impaired oral hygiene and saliva stasis in the oral cavity that may all contribute to the periodontal diseases. These factors are enough to require close dental follow up and should not be compared to children with normal motor skills and who are able to control saliva swallowing. In our 36 month follow up we did not observe any increase in dental or periodontal problems, as reported by other authors.^4^

Three children had PT duct unilateral fistulas, and this did not impact procedure success, having seen that saliva reduction brought about by SM gland excision was enough to control sialorrhea. Two children developed a salivary retention cyst in the jugal region, adjacent to the PT duct closure; we performed punction and drainage, without recurrence.

The effort children with neuropathy put up in order to control sialorrhea may even impair their development of other oral motor skills, such as speech and mastication6. We have observed the acquisition or the very improvement of oral motor skills in 32% of the patients under speech therapy follow up in the first three months after the procedure, sucking through a straw, more efficient mastication and improvement in speech intelligibility.

## CONCLUSION

The bilateral resection of submandibular glands together with parotid ducts ligature is a safe and efficient technique to be carried out in children with motor oral incapacity, preventing saliva oral escape, with low complications rate.
